# Analyse steigender Behandlungskosten bei erhöhtem BMI von Patienten mit proximaler Femurfraktur

**DOI:** 10.1007/s00113-022-01187-8

**Published:** 2022-05-23

**Authors:** Alexander Gutwerk, Michael Müller, Moritz Crönlein, Chlodwig Kirchhoff, Peter Biberthaler, Dominik Pförringer, Karl Braun

**Affiliations:** 1 Klinik für Orthopädie, Unfall‑, Hand- und Plastische Chirurgie, DIAKO Krankenhaus, Knuthstr. 1, 24939 Flensburg, Deutschland; 2 Klinik und Poliklinik für Unfallchirurgie, Klinikum rechts der Isar, Technische Universität München, München, Deutschland; 3 Centrum für Muskuloskeletale Chirurgie, Charité Universitätsmedizin Berlin, Berlin, Deutschland

**Keywords:** Aufenthaltsdauer, Gesundheitssystem, Endoprothetik, Osteosynthese, Adipositas, Duration of stay, ALOS (average length of stay), Body weight, Internal fixation, Obesity

## Abstract

Die proximale Femurfraktur ist eine Frakturentität mit ansteigender Prävalenz, ein Effekt, welcher durch die zunehmende Alterung der Allgemeinbevölkerung noch zusätzlich verstärkt werden wird. In der Summe stellt sie sowohl die Operateure als auch das deutsche Gesundheitssystem als Ganzes vor Herausforderungen. Zudem führt ein stetiges Ansteigen des BMI in der Bevölkerung zu immer mehr adipösen Patienten mit proximaler Femurfraktur, was dann durch komplexere Operationen, längere Liegezeiten und eine wachsende Zahl postoperativer Komplikationen wiederum zu Herausforderungen führt.

Ziel der vorliegenden Arbeit ist es, den monetären Aspekt dieser Entwicklung zu beleuchten. Hierzu wurden retrospektiv 950 Patientenfälle mit proximaler Femurfraktur analysiert. Hierbei konnte gezeigt werden, dass mit steigendem BMI höhere Kosten pro Fall entstanden (10.452 €, 11.505 €, 12.085 € bzw. 13.681 € für BMI < 18,5 kg/m^2^, BMI = 18,5–24,9 kg/m^2^, BMI = 25,0–29,9 kg/m^2^ bzw. BMI ≥ 30,0 kg/m^2^), da sowohl die Operationszeit als auch die Zeit des stationären Aufenthalts relevant verlängert waren.

## Abstract (en)

Driven by the aging population, the proximal femoral fracture is a rising fracture entity, challenging trauma surgeons as well as the German healthcare system as a whole. The rising average age of the population is accompanied by a rising BMI in the German population, resulting in longer operation times, longer average length of stay (ALOS) as well as more postoperative complications.

The aim of this study was to demonstrate the economic correlation between body mass index and hospital costs. The retrospective analysis of 950 patient cases suffering from a proximal femoral fracture resulted in the finding of rising treatment costs being positively associated with rising BMI due to longer operation times and longer length of hospitalization (10,452 €, 11,505 €, 12,085 € and 13,681 € for patients with BMI < 18.5 kg/m^2^, BMI = 18.5–24.9 kg/m^2^, BMI = 25.0–29.9 kg/m^2^ and BMI ≥ 30.0 kg/m^2^, respectively).

## Hintergrund

Die proximale Femurfraktur stellt in zunehmendem Maße nicht nur die behandelnden Operateure, sondern auch das Gesundheitssystem als Ganzes vor eine Herausforderung. Sie ist eine der Schlüsselfrakturen des alten Patienten und wies bereits 2006 eine Inzidenz von ca. 100.000 Patienten/Jahr auf [9], die dann zunehmend von 108.000 Fällen (2014) auf 118.000 Fälle im Jahr 2017 anstieg [10]. Entsprechend zeigen die letzten Studien bei über 65-jährigen eine Inzidenz von 600 bis 900 proximalen Femurfrakturen/100.000 Einwohner und Jahr [13]. Somit stellen proximale Femurfrakturen, gemäß der Erhebung der „*Rotterdam Study*“, bei Patienten ab 55 Jahren die häufigste Extremitätenfraktur bei Männern und die zweithäufigste bei Frauen dar [20]. Problematisch hierbei ist es, dass der steigenden Fallzahl dieser häufig multimorbiden Patienten eine sinkende Anzahl an versorgenden Kliniken gegenübersteht – ein durch das Gesundheitssystem hausgemachtes Problem, da monetäre Kompensationsmechanismen für Krankenhäuser, die diese aufwendige Versorgung vorhalten und entsprechend steigende Kosten hierfür tragen, nicht existieren [10].

Parallel hierzu zeigt sich eine Zunahme von Übergewicht in der Gesellschaft. Waren 1985 noch jeweils 16,2 % der deutschen Männer und Frauen adipös, so waren es 2002 bereits 22,5 % der Männer und 23,3 % der Frauen. In der letzten bundesweiten Erhebung des Robert Koch-Instituts wurde die Prävalenz der Adipositas („starkes Übergewicht“) mit 23 % angegeben, die Prävalenz des Übergewichts mit rund 60 % [18].

Die Kombination eines erhöhten Body-Mass-Index und proximaler Femurfraktur kann sich nun wiederum negativ auf die Mortalität und Morbidität der Patienten auswirken [11]. Die zusätzlichen operativen Herausforderungen reichen von der erschwerten Zugangspräparation, der schwierigeren Positionierung der Endoprothese über den aufwendigeren Wundverschluss bis hin zur gesteigerten Komplikationsrate durch Wundinfektionen und Blutungen. Die Operationszeit und der stationäre Aufenthalt (ALOS) verlängern sich ebenfalls [3, 4, 16, 21–24].

Problematisch ist hierbei neben den erhöhten Anforderungen an den behandelnden Chirurgen aus gesundheitsökonomischer Sicht auch das Problem der fehlenden Refinanzierung dieser Herausforderungen. Ziel dieser Arbeit war es daher, die Mehrkosten bei der Versorgung von Patienten mit proximaler Femurfraktur und hohem BMI zu analysieren, um einen ersten Anhaltspunkt für eine mögliche Diskussion über eine Rückvergütung der entstehenden Kosten zu liefern.

## Material und Methoden

Die Studie wurde dem Ethikkomitee der medizinischen Fakultät des Klinikums Rechts der Isar (TU München) vorgelegt und unter der Studiennummer 409/15s akzeptiert. Retrospektiv wurden für den Zeitraum von Dezember 2003 bis Juni 2015 950 Patienten eingeschlossen, welche bei proximaler Femurfraktur in der Klinik für Unfallchirurgie des Klinikums Rechts der Isar operiert worden waren. Diese wurden im klinikeigenen Computerarchiv (SAP) anhand der ICD-10-Codes S72.0–S72.2 ermittelt. Ausgeschlossen wurden alle konservativ behandelten Patienten sowie Patienten mit Fraktur des Femurkopfes. Polytraumatisierte Patienten und Patienten mit periprothetischen oder pathologischen Frakturen (z. B. Prothesenwechsel, pathologische Frakturen bei tumoröser Grunderkrankung) wurden von der Analyse ausgeschlossen. Die Daten wurden von der Aufnahme bis zu Entlassung des Patienten erhoben. Eine regelhafte Nachuntersuchung erfolgte nicht.

### Body-Mass-Index

Der BMI wurde anhand der von der WHO aufgestellten Kriterien eingeteilt ([25]; Tab. 1).

**Table 1:** 

BMI < 18,5 kg/m^2^	Untergewicht	UW
BMI 18,5–24,9 kg/m^2^	Normalgewicht	NW
BMI 25,0–29,9 kg/m^2^	Übergewicht/Präadipositas	OW
BMI ≥ 30,0 kg/m^2^	Adipositas (Grade I–III)	OB

*BMI* Body-Mass-Index; *NW* „Normal weight“ (Normalgewicht); *OB* „Obese“ (Adipositas [Grade I–III]); *OW* „Overweight“ (Übergewicht/Präadipositas); *UW* „Underweight“ (Untergewicht)

### Operative Therapie

Frakturen im Bereich des Schenkelhalses wurden mittels dynamischer Hüftschraube (DHS Dynamic Hip System; Fa DePuy Synthes GmbH, Oberdorf, Schweiz) oder endoprothetisch mittels Totalendoprothese oder Hemiendoprothese (Fa. Zimmer Biomet, Warsaw, IN, USA) je nach den jeweiligen Ansprüchen und Voraussetzungen des Patienten versorgt. Die endoprothetische Versorgung erfolgte unter Verwendung des anterolateralen Watson-Jones-Zuganges.

Der überwiegende Teil von Frakturen des Trochanterbereichs wurde unter Verwendung eines Extensionstisches über einen lateralen Zugang mittels Marknagelsystemen versorgt (Proximal Femoral Nail Antirotation System; Fa. Synthes GmbH, Oberdorf, Schweiz, oder TRIGEN INTERTAN Intertrochanteric Antegrade Nail System; Fa. Smith and Nephew Inc., Cordova, TN, USA), eine kleine Zahl von Patienten wurde mittels dynamischer Hüftschraube (DHS Dynamic Hip System; Fa. DePuy Synthes GmbH, Oberdorf, Schweiz) versorgt.

Endoprothetisch und mittels Marknagelung versorgten Patienten wurde die umgehende Vollbelastung erlaubt, während diese bei den mit DHS versorgten Patienten auf 15 kg eingeschränkt wurde. Die Range of Motion (RoM) wurde postoperativ bei allen Patienten auf 90°-Hüftflexion limitiert.

Die Operationszeit, definiert als Schnitt-Naht-Zeit, wurde der elektronischen Dokumentation der Op-Pflege entnommen (SAP) und mittels dieser konsekutiv die Kosten der operativen Versorgung über den OP-Minuten-Preis für das Klinikum rechts der Isar von 12,48 €/min [17] kalkuliert.

### Stationärer Aufenthalt

Die Dauer des stationären Aufenthalts wurden anhand des im elektronischen Krankenhausinformationssystem (SAP) dokumentierten Aufnahme- und Entlasszeitpunktes errechnet. Die Kosten ergaben sich anhand dieser Aufenthaltsdauer in Tagen, multipliziert mit den durchschnittlichen Kosten eines Aufenthaltstages auf der Station.

Die Kosten pro Zeiteinheit für den stationären Aufenthalt divergieren in Deutschland ebenso wie deren Kalkulationsgrundlage. Am Klinikum rechts der Isar werden diese anhand der ex post kalkulierten Kosten im vorausgegangenen Kalkulationsjahr pro Patient und Zeiteinheit kalkuliert. Hierbei wird aufgrund der Kostenstruktur eines Supramaximalversorgers mit 53 Cent/min, d. h. 763,20 €/Tag an Aufenthaltskosten auf der traumatologischen Normalstation kalkuliert.

In einem Fall konnte aufgrund fehlerhafter Dokumentation die Liegezeit eines Patienten (97 Jahre, weiblich; BMI-Gruppe NW mit 24,2 kg/m^2^; Schnitt-Naht-Zeit 37 min) nicht sicher bestimmt werden, sodass sich die Grundgesamtheit für die stationäre Liegedauer auf 949 Patienten bezieht. Hieraus ergibt sich auch eine Differenz in der Summation der durchschnittlichen Kosten von Operation und Aufenthalt zu den errechneten Durchschnittsgesamtkosten.

### Statistische Analyse

Die statistische Analyse erfolgte unter Verwendung von SPSS (Version 22, IBM SPSS Statistics for Windows; Fa. Armonk, NY, USA). Zur Beschreibung kontinuierlicher Variablen wurde der Mittelwert mit Standardabweichung angegeben. Für kontinuierliche Variablen wurde der Kruskal-Wallis-Test, für dichotome der Chi-Quadrat Test angewandt, als Signifikanzlevel wurde *p* < 0,05 bestimmt.

## Ergebnisse

**Figure 1:**
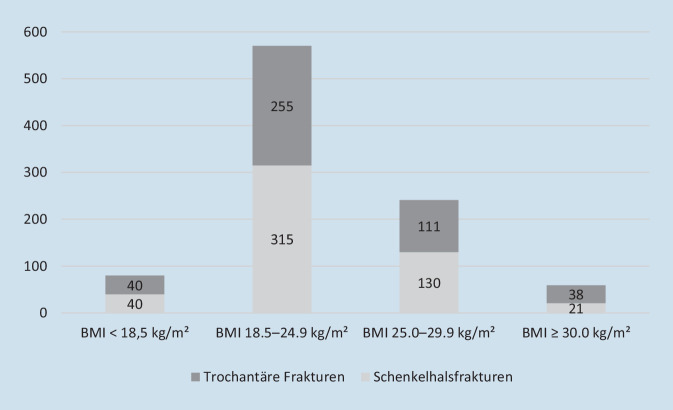

Es wurden die Daten von 950 Patienten analysiert, hiervon waren 80 untergewichtig, 570 in der Gruppe des Normalgewichts, 241 übergewichtig und 59 adipös. Dabei hatten 506 Patienten eine Schenkelhalsfraktur und 444 eine Fraktur des Trochanterbereichs erlitten (Abb. 1).

**Figure 2:**
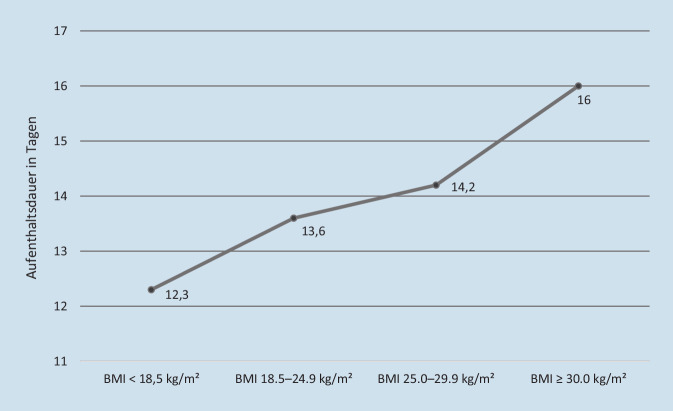

Der stationäre Aufenthalt wurde im Durschnitt mit Kosten von 10.509,48 € (±6809,41 €) beziffert. Die Aufenthaltsdauer – und mit ihr die Kosten für den stationären Aufenthalt – stiegen mit steigendem BMI nahezu linear an (Abb. 2; Tab. 2).

**Table 2:** 

BMI-Gruppe	ALOS in Tagen	Standardabweichung	Kosten in €	Prozentual
UW	12,3	±4,8	9387,36	90,4
NW	13,6	±7,8	10.379,52	100
OW	14,2	±11,7	10.837,44	104,4
OB	16,0	±9,7	12.211,20	117,6

*NW* „Normal weight“ (Normalgewicht); *OB* „Obese“ (Adipositas [Grade I–III]); *OW* „Overweight“ (Übergewicht/Präadipositas); *UW* „Underweight“ (Untergewicht)

Bei einer reinen Kalkulation der Aufenthaltskosten nach den 4 BMI Gruppen ergibt sich folgende Kostenkorrelation.

**Figure 3:**
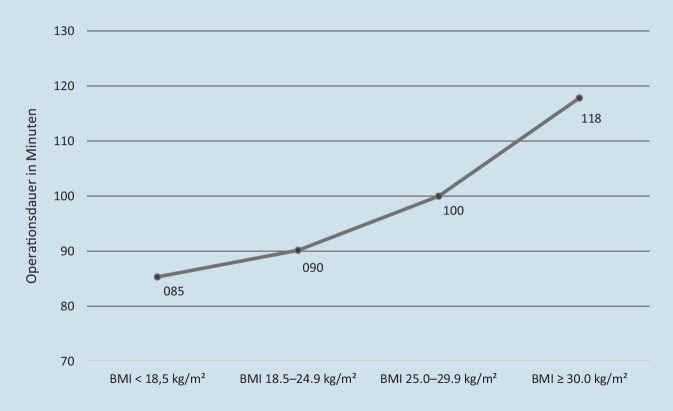

Ebenso verlängerte sich die Operationszeit für die Frakturversorgung mit steigendem BMI (Abb. 3), was parallel zu einer kontinuierlichen Kostensteigerung von rund 1065 € in der untergewichtigen Gruppe auf über 1470 € in der adipösen Gruppe führte (Tab. 3). Im Durchschnitt kostete die operative Versorgung 1172,36 € (±517,46 €).

**Table 3:** 

BMI-Gruppe	Operationszeit in Minuten	Standardabweichung	Operationskosten in Euro	Prozentual
UW	85,3375	±42,9	1065,01	94,6
NW	90,16637	±38,2	1125,28	100
OW	99,98755	±39,9	1247,84	110,9
OB	117,8103	±61,5	1470,27	130,7

*NW* „Normal weight“ (Normalgewicht); *OB* „Obese“ (Adipositas [Grade I–III]); *OW* „Overweight“ (Übergewicht/Präadipositas); *UW* „Underweight“ (Untergewicht)

**Figure 4:**
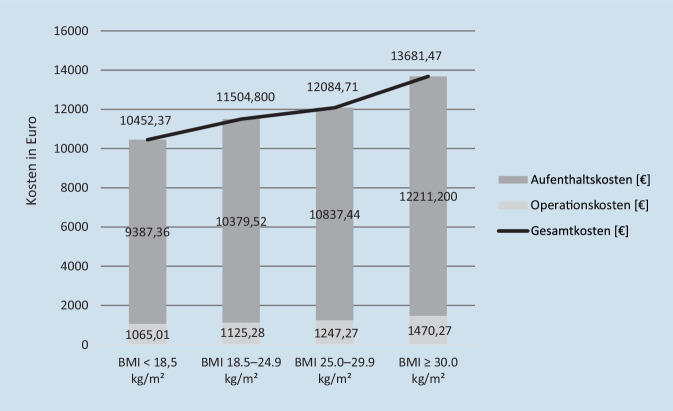

In der Summe ergaben sich durchschnittliche Fallkosten von 11.682,59 € (±6895,99 €), bei einem stetigen Anstieg mit steigendem BMI. Hierzu trugen sowohl die gesteigerten Operationskosten als auch die gesteigerten Aufenthaltskosten bei zunehmendem BMI bei (Abb. 4).

## Diskussion

Im Rahmen der Kostenanalyse zeigt sich ein erhöhter Kostenaufwand bei Patienten mit erhöhtem BMI und proximaler Femurfraktur. Diese Mehrkosten werden aktuell nicht durch das „German-Diagnosis-Related-Groups“(G-DRG)-System in ausreichender Höhe kompensiert, obwohl sich eine nahezu lineare Kostensteigerung (Abb. 4) in der Behandlung von Patienten mit proximaler Femurfraktur bei erhöhtem BMI nachweisen lässt. Aufgrund des demografischen Wandels und der erwarteten Zunahme an proximalen Femurfrakturen und Adipositas stellt dies eine große finanzielle Belastung der behandelnden Kliniken dar. Kernkomponenten in einer solchen Betrachtung sind die Kosten der operativen Versorgung sowie die Kosten des stationären Aufenthalts [1].

Die auf diese Weise von uns approximierten Gesamtkosten von 11.682,59 € liegen über den bereits für Deutschland publizierten Daten [1, 8]. Aigner et al. [1] beziffern die Gesamtkosten für die Behandlung von Patienten mit proximaler Femurfraktur am Universitätsklinikum Gießen/Marburg im Durchschnitt auf etwa 8853 € (±5676 €), davon die Kosten des stationären Aufenthalts auf durchschnittlich 5828 € und die Operationskosten auf durchschnittlich 1972 €. Hanstein et al. [8] berichten in ihrer Studie für elektive Hüft-TEP-Versorgungen (unzementierte TEP vs. Hybrid-TEP) an der Orthopädischen Universitätsklinik Rostock von durchschnittlichen Operationskosten von 3370,07 € vs. 3308,30 € (exkl. der Sachkosten gemäß Listenpreis 2230,25 € vs. 2038,05 € ergeben sich also Operationskosten von 1139,82 € vs. 1270,25 €). Die Gesamtkosten beliefen sich auf 6793,52 € für die unzementierte und 6605,38 € für die hybride TEP. Hierbei gilt es, im Hinterkopf zu behalten, dass es sich um elektiv geplante Hüftendoprothesen handelt und nicht das gemischte Patientengut der akuten proximalen Femurfraktur, welches wir in unserer Erhebung betrachten. Somit waren ex ante höhere Kosten in unserer Erhebung zu erwarten gewesen. Im Vergleich zu Aigner et al. [1] zeigen sich unsere Kosten für den stationären Aufenthalt deutlich erhöht, jedoch bei einer ähnlichen Belegungsdauer (13,7 ± 8,9 Tage in unserer Erhebung gegenüber 14 ± 6 Tagen).

In unserer getrennten Analyse beider Kostenpunkte konnten wir eine Verlängerung der operativen Versorgungzeiten durch einen steigenden BMI mit einem monetären Mehraufwand in Verbindung bringen und zeigen, dass die kalkulierten Operationskosten durch verlängerte Operationszeiten mit steigendem Patienten-BMI stetig von ca. 1065 € (BMI unter 18,5 kg/m^2^) auf rund 1470 € (BMI über 30 kg/m^2^) in der Gruppe der adipösen Patienten ansteigen. Diese Verlängerung der Operationszeiten bei steigendem BMI zeigt sich auch in anderen Studien [15, 19]. Mitverantwortlich scheint die größere Fettgewebeschicht zu sein, welche die Operationszeit mit zunehmendem Patientenumfang fast linear um 20 min je „1 cm Zugangstiefe“ verlängert [15].

Die Arbeitsgruppe um Klopfer beschreibt für das Jahr 2017 eine Inzidenz von 118.000 Schenkelhalsfrakturen in Deutschland pro Jahr [10]. Gleichzeitig gibt das Robert Koch-Institut (RKI) die Prävalenz der Adipositas mit 23 % an, die Prävalenz des Übergewichts (inkl. Adipositas) mit rund 60 % [18].

Die Kombination dieser Zahlen zeigt bei rund 120.000 Fällen/Jahr [10] und 23 % adipösen sowie weiteren 37 % übergewichtigen Patienten [18] eine kalkulierte Kostensteigerung von rund 85,9 Mio. € pro Jahr (70,9 Mio. € durch verlängerte Liegedauer und 15,0 Mio. € durch verlängerte Operationszeiten) für das deutsche Gesundheitssystem. Die potenziellen Folgekosten aufgrund gesteigerter Komplikationsraten sind zu dieser Kalkulation noch zu addieren, sowie darüber hinaus die zu erwartende weitere Zunahme der Fallzahlen [10].

Da dieser Kostenanstieg im derzeitigen DRG-System nicht adäquat abgebildet wird, jedoch aufgrund der zunehmenden Überalterung und der breiten Gewichtszunahme von dessen Aggravation auszugehen ist, bietet sich eine Diskussion über die Einbeziehung des BMI in die Vergütungsstrukturen der Kliniken an.

In Deutschland wird seit der Einführung des Gesundheitsreformgesetzes 2000 über das fallpauschalenbasierte G‑DRG-System abgerechnet. Eine monetäre Mehrvergütung für Patientin mit erhöhtem BMI ist in diesem nur bedingt vorgesehen. So schlägt sich für die ICD-10-Diagnosen S72.00–S72.2 erst eine BMI-Erhöhung über 40 kg/m^2^ in einer CCL von 2 nieder, also erst bei Erreichen der höchsten Stufe der WHO-Klassifikation der Adipositas (WHO Adipositas Grad III). Für BMI-Werte unter 40 kg/m^2^ ergibt sich durch die z. T. bereits erhebliche Adipositas und damit einhergehende Einschränkungen keine Steigerung der DRG. In unserer 950 Patienten umfassenden Kohorte ergab sich somit für lediglich 5 Patienten eine potenzielle Steigerung der DRG (durch die Steigerung der CCL), obwohl wir zeigen konnten, dass die Behandlungskosten bereits in den Gruppen mit einem BMI-Wert über 25 kg/m^2^ erhöht waren und somit eine Rückvergütung dieser Mehrkosten in 300 Fällen unserer Kohorte wünschenswert gewesen wäre. Durch eine Verkürzung der durchschnittlichen Verweildauer bei primärer Hüft-TEP-Implantation in Deutschland von 13,6 Tagen im Jahr 2009 auf 11,8 (2014) und weiter bis auf 10,2 Tage im Jahr 2017 [2] konnte der Effekt erzeugt werden, dass in Deutschland im weltweiten Vergleich endoprothetische Operationen relativ kostengünstig durchgeführt werden [5, 12]. Allerdings ergeben sich diese Einsparungen durch eine reduzierte Grenzverweildauer, welche nun bei sich veränderndem Patientenkollektiv an ihre Optimierungsgrenzen stößt.

Wie von Nyszkiewicz anschaulich beschrieben, führen diese verkürzte Verweildauer und jede andere Einsparung mit einer 2‑jährigen Verzögerung durch die Neubewertung der DRG zu einer Schlechterbewertung der DRG bzw. einer Anpassung der kalkulierten Verweildauer und somit einer zukünftigen Erlösminderung, welche die Kliniken zu neuen Sparmaßnahmen drängen [14]. Dies führt zwangsläufig zu einer Qualitätsverschlechterung und einer Patientenselektion in „gewinnversprechende vs. verlustbehaftete Fälle“. Dass wirtschaftliche Faktoren einen zunehmend wichtigen Faktor darstellen und Ärzten mehr und mehr eine ökonomische Logik aufgezwungen wird, ist bekannt [6, 26]. Im Bereich der elektiven Endoprothetik ist bereits ein Trend erkennbar, sich auf ein gesundes Patientenkollektiv zu konzentrieren, um finanzielle Verluste zu vermeiden [7]. Diese Vorselektion ist jedoch in der meist notfälligen Versorgung proximaler Femurfrakturen nicht durchführbar und betont die dringende Notwendigkeit einer Kostenanpassung in diesem Kollektiv.

Anhand unserer Studie wird es ersichtlich, dass die anfallenden Mehrausgaben durch den erhöhten Aufwand der Versorgung proximaler Femurfrakturen bei Patienten mit erhöhtem BMI im DRG-System abgebildet werden müssen. Dies steht in Analogie zu den Forderungen der Kollegen im geriatrischen Sektor, um die höheren Kosten der Therapie bei Osteoporose, multiplen Vorerkrankungen sowie Schwierigkeiten bei Mobilisation und Rehabilitation abzubilden.

Die Ergebnisse dieser Studie unterliegen einigen Limitationen. Es wurden ausschließlich Patienten aus einem universitären, überregionalen Traumazentrum eingeschlossen. Die Vorhaltungskosten sind hier im Vergleich zu kleineren Häusern in der Regel höher.

Die Datenerhebung erfolgte retrospektiv; eine regelhafte Nachuntersuchung erfolgte nicht.

Die operative Versorgung erfolgte durch verschiedene Operateure in verschiedenen Stadien der Aus- und Weiterbildung. Eine Analyse nach Operateur erfolgte nicht.

Für die Kostenanalyse wurden lediglich die größten Kostenpunkte (Kosten der operativen Versorgung und des stationären Aufenthalts) berücksichtigt, Folgeeingriffe, Komplikationsraten oder der Mehrverbrauch an Materialien wurden nicht berücksichtigt, sondern lediglich die Operationszeit der Primärimplantation.

Die zur Kalkulation der Operationskosten verwendeten Zahlen (Euro/Operationsminute) wurden bereits im Jahr 2017 publiziert und stammen nicht aus diesem Jahr. Eine in der Zwischenzeit eingetretene, wesentliche Änderung ist nicht zu erwarten, jedoch nicht sicher auszuschließen. Der publizierte Zeitraum von Dezember 2003 bis Juni 2015 liegt bereits einige Jahre in der Vergangenheit. Aufgrund von veränderten Ablaufstandards und Implantaten konnte er nicht wie gewünscht bis 2020 oder 2021 verlängert werden.

Die Standardabweichung der ermittelten Kosten zeigte sich im Verhältnis zum Mittelwert als sehr groß. Diesen Umstand bedauern wir, jedoch zeigen die evaluierten Zahlen einen klaren Trend. Es sollte mittels größer angelegter Studien versucht werden, diesen statistischen Makel zu beheben und die vorliegenden Ergebnisse damit einer erneuten Prüfung zu unterziehen.

## Fazit für die Praxis

- Aufgrund der demografischen Entwicklung sowie der kalorischen Versorgungslage kommen in Europa mehr und mehr übergewichtige Patienten vor.
- Auf die Kliniken kommen in diesem Kontext neue Herausforderungen und damit verbundene Kosten zu.
- Die Kostenträger werden auf die Problematik und den steigenden Ressourcenverbrauch hingewiesen.
- Mittelfristig wird der BMI in die DRG-Vergütung einfließen müssen, um den sich verändernden Rahmenbedingungen gerecht zu werden.

## Abkürzungen

ALOS: „Average length of stay“
BMI: Body-Mass-Index
CCL: „Complication or comorbidity level“
G‑DRG: German Diagnosis Related Groups
ICD‑10: International Statistical Classification of Diseases and Related Health Problems – 10. Ausgabe
NW: „Normal weight“ (Normalgewicht)
OB: „Obese“ (Adipositas (Grade I–III))
OW: „Overweight“ (Übergewicht/Präadipositas)
RKI: Robert Koch-Institut
RoM: „Range of Motion“
SAP: Krankenhaussoftware der SAP SE (Systemanalyse Programmentwicklung)
TEP: Totalendoprothese
UW: „Underweight“ (Untergewicht)
